# Successful Management of Advanced Sinonasal Undifferentiated Carcinoma Using Concurrent Chemoradiotherapy With Simultaneous Integrated Boost and Intensity-Modulated Radiation Therapy: A Case Report

**DOI:** 10.7759/cureus.85170

**Published:** 2025-06-01

**Authors:** Sawa Kono, Ryotaro Ando, Munenaga Nakamizo, Yoji Nagashima, Yaichiro Hashimoto

**Affiliations:** 1 Radiation Oncology, Tokyo Women's Medical University, Tokyo, JPN; 2 Otorhinolaryngology-Head and Neck Surgery, Tokyo Women's Medical University, Tokyo, JPN; 3 Surgical Pathology, Tokyo Women's Medical University, Tokyo, JPN

**Keywords:** concurrent chemoradiotherapy, intensity-modulated radiation therapy, simultaneous integrated boost, sinonasal undifferentiated carcinoma, vision restoration

## Abstract

Sinonasal undifferentiated carcinoma (SNUC) is a rare and aggressive malignancy with a poor prognosis, often presenting at an advanced stage owing to nonspecific symptoms and delayed diagnosis. Although the understanding of the histological and immunohistochemical characteristics of SNUC has improved, treatment options remain limited, and patient outcomes are suboptimal.

We present a case of advanced SNUC originating in the left nasal cavity of a 53-year-old man, with visual-field impairment as the primary symptom. Based on imaging and histopathological findings, including a high Ki-67 index, as well as immunohistochemical positivity for cytokeratin and CD56, the patient was diagnosed with Stage IVB SNUC. Given the extent of local invasion, surgical resection was infeasible, and concurrent chemoradiotherapy (CCRT) was selected as the primary treatment. The regimen consisted of platinum-based chemotherapeutic agents, including cisplatin and etoposide (PE), combined with intensity-modulated radiation therapy using the simultaneous integrated boost technique to optimize tumor targeting while sparing critical structures such as the optic nerve. Complete tumor resolution was confirmed by post-treatment magnetic resonance imaging, with no evidence of recurrence or metastasis at the three-year follow-up. The patient reported significant improvement in vision, including the resolution of left hemianopia, which contributed to an enhanced quality of life. This case highlights the efficacy of CCRT in achieving durable disease control and functional recovery in patients with advanced SNUC. These findings underscore the potential of precision radiotherapy techniques and multimodal strategies for managing this challenging malignancy.

## Introduction

Sinonasal undifferentiated carcinoma (SNUC) is a rare and aggressive malignancy arising from the epithelium of the nasal cavity and paranasal sinuses. First characterized by Frierson et al. in 1986 [1], SNUC accounts for fewer than 1% of all head and neck cancers [2,3]. Due to its nonspecific early symptoms, such as nasal obstruction and epistaxis, SNUC is often diagnosed at an advanced stage, frequently involving the skull base and orbit. Untreated, median survival is approximately 12 months, and no standardized treatment protocol has been established [3,4]. The diagnosis of SNUC requires careful histopathological evaluation to distinguish it from other high-grade sinonasal tumors, such as olfactory neuroblastoma and neuroendocrine carcinoma. Immunohistochemistry and the exclusion of different entities are essential for accurate diagnosis. Given the aggressive nature of SNUC and its tendency toward extensive local invasion and distant metastasis, multimodal therapy is typically required. Although surgery followed by radiotherapy is considered standard for resectable cases, in many instances, complete resection is not feasible due to anatomical constraints [5]. In such cases, chemoradiotherapy serves as an alternative. The advent of precision radiotherapy techniques, such as intensity-modulated radiation therapy (IMRT) and simultaneous integrated boost (SIB), has allowed for the delivery of high-dose radiation to target volumes while minimizing exposure to critical structures [6]. Herein, we present unresectable, locally advanced SNUC successfully treated with definitive concurrent chemoradiotherapy using IMRT with SIB. This case contributes to the limited but growing body of evidence supporting non-surgical curative approaches in the management of SNUC.

Aggressive multimodal treatment, combining surgery, platinum-based chemotherapy, and radiotherapy, is essential for locoregional control and long-term survival [4]. Patients undergoing primary surgical resection followed by postoperative radiotherapy typically demonstrate better outcomes than those receiving radiotherapy alone [5].

Although understanding of the histological and immunohistochemical characteristics of SNUC has improved, treatment options remain limited, and patient outcomes are suboptimal [2]. To date, no standardized treatment protocol has been established. Chemotherapy with PE therapy, based on the biological similarities between SNUC and small-cell lung cancer, has been reported as a potential therapeutic option [6].

Herein, we present a case of SNUC originating in the left nasal cavity, in which tumor resolution was achieved with chemoradiotherapy, and visual-field defects were alleviated.

## Case presentation

**Figure 1:**
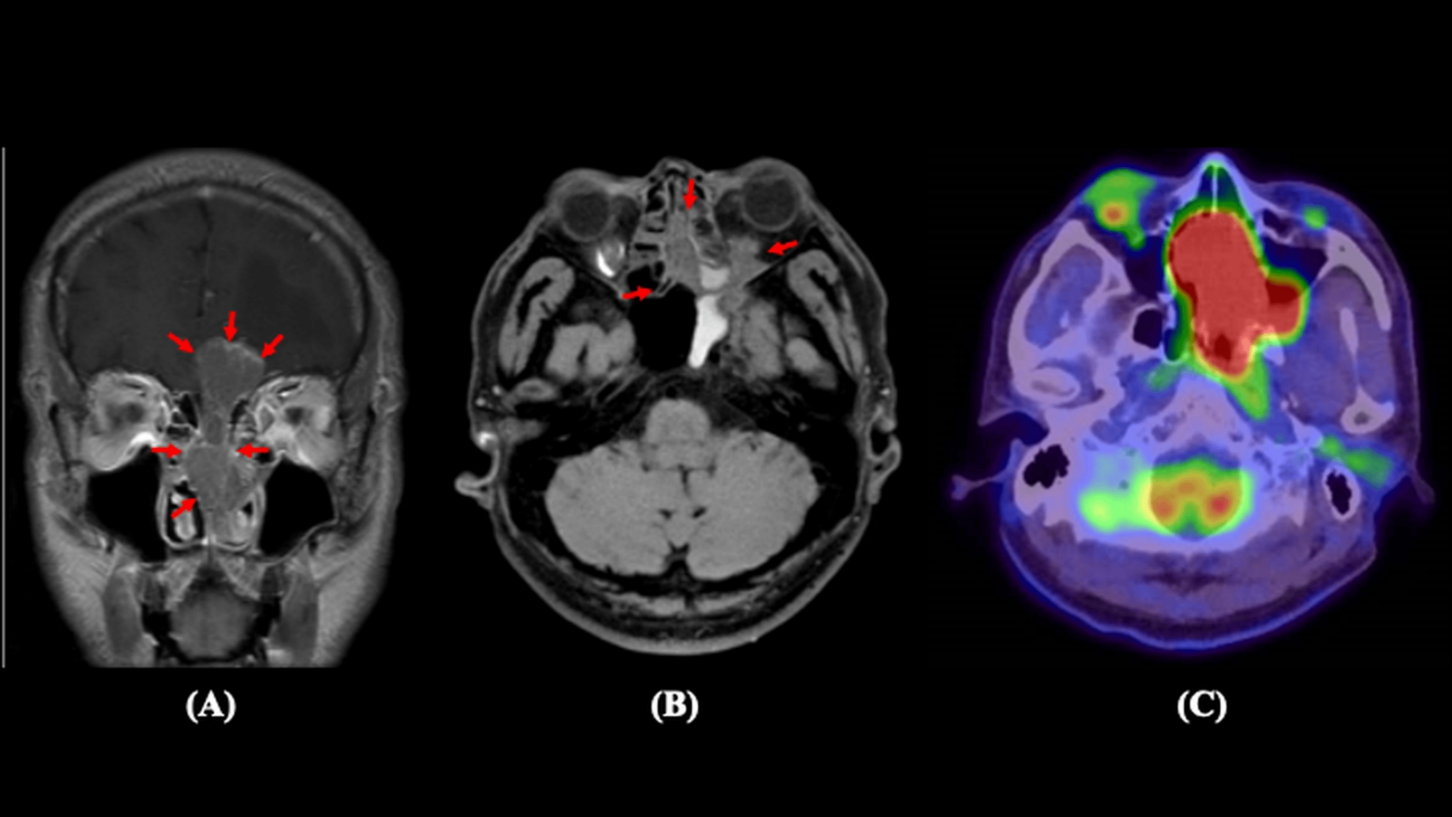
MRI and PET/CT imaging of the sinonasal tumor (A) Coronal and (B) axial T1-weighted MRI images with gadolinium contrast enhancement demonstrate a well-enhancing tumor with extensive invasion into the anterior cranial base, bilateral orbits, and adjacent paranasal sinuses (red arrows). (C) The FDG-PET/CT image shows intense FDG uptake in the primary tumor, consistent with high metabolic activity.

A 53-year-old man visited our clinic with a two-month history of left visual-field impairment. Ophthalmological examination revealed left inferior hemianopia. Computed tomography (CT) and gadolinium-enhanced T1-weighted magnetic resonance imaging (MRI) showed a tumor extending from the left nasal cavity to the anterior cranial base without evidence of regional or distant metastases (Figures 1A, 1B). On MRI, the tumor measured approximately 3.8 × 2.5 × 2.3 cm. Fluorodeoxyglucose positron emission tomography (FDG-PET) demonstrated intense uptake localized to the tumor, with no abnormal accumulation in regional lymph nodes or distant organs (Figure 1C).

**Figure 2:**
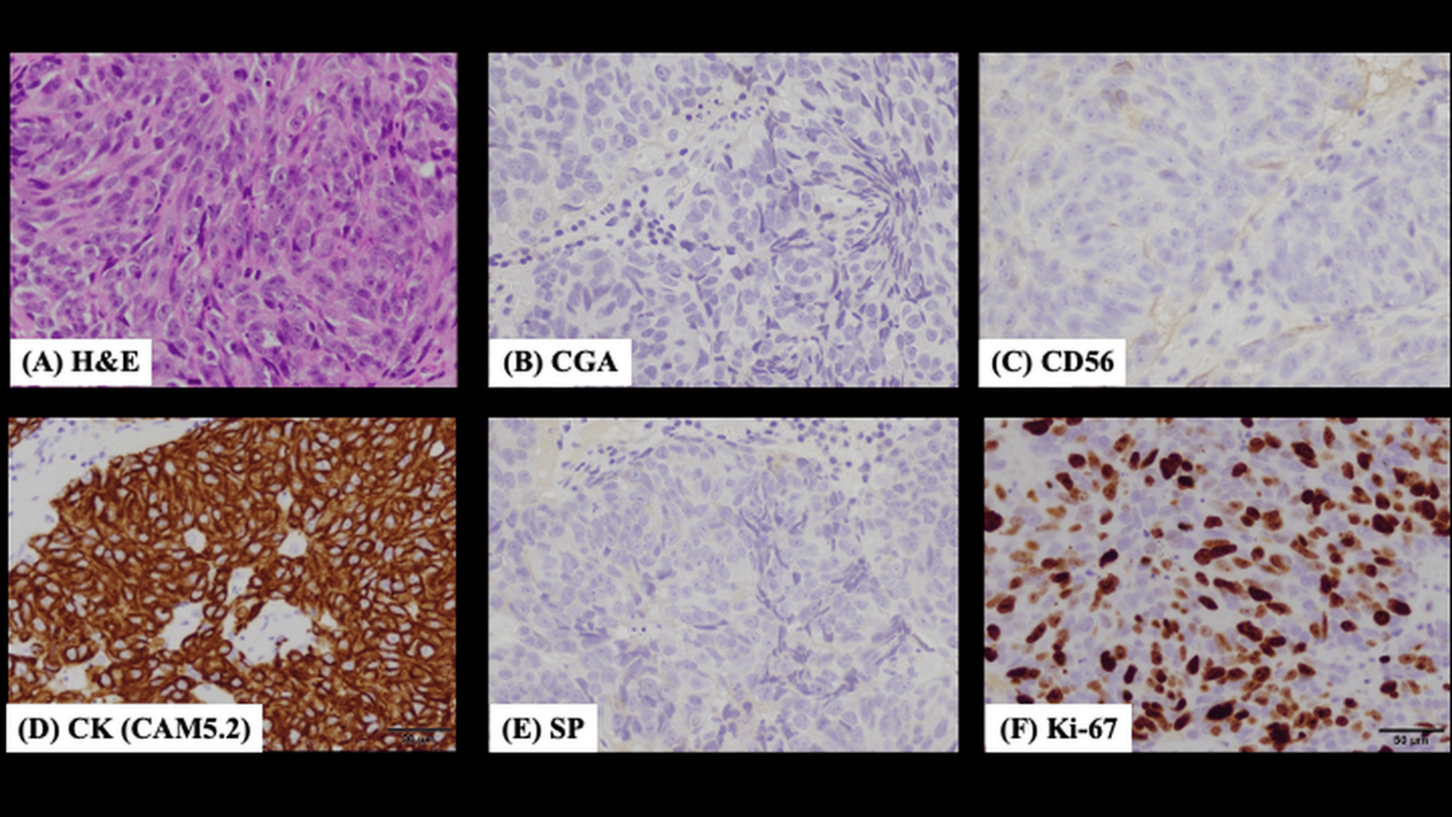
Histopathological and immunohistochemical findings of the biopsy specimen (A) Hematoxylin and eosin (H&E) staining showing sheets of undifferentiated tumor cells; (B) Immunohistochemistry for chromogranin A is negative; (C) CD56 shows strong membranous positivity; (D) Cytokeratin (CAM5.2) demonstrates diffuse positivity; (E) Synaptophysin (SP) is negative; (F) Ki-67 labeling index is approximately 60%, indicating high proliferative activity All images were taken at an original magnification of 400×.

Histopathological evaluation of the biopsy specimen (Figure 2) confirmed an undifferentiated carcinoma, and immunohistochemical analysis revealed positivity for cytokeratin (CK) and CD56, with a Ki-67 labeling index of 60%, indicating high proliferative activity. Hematoxylin and eosin staining revealed a nest-like arrangement of tumor cells. Results of tests for chromogranin and synaptophysin were negative, ruling out a neuroendocrine tumor, whereas CK positivity confirmed the epithelial origin of the malignancy. Based on these findings, the patient was diagnosed with SNUC. The tumor was classified as T4bN0M0, Stage IVB, according to the 8th edition of the TNM classification system [7].

**Figure 3:**
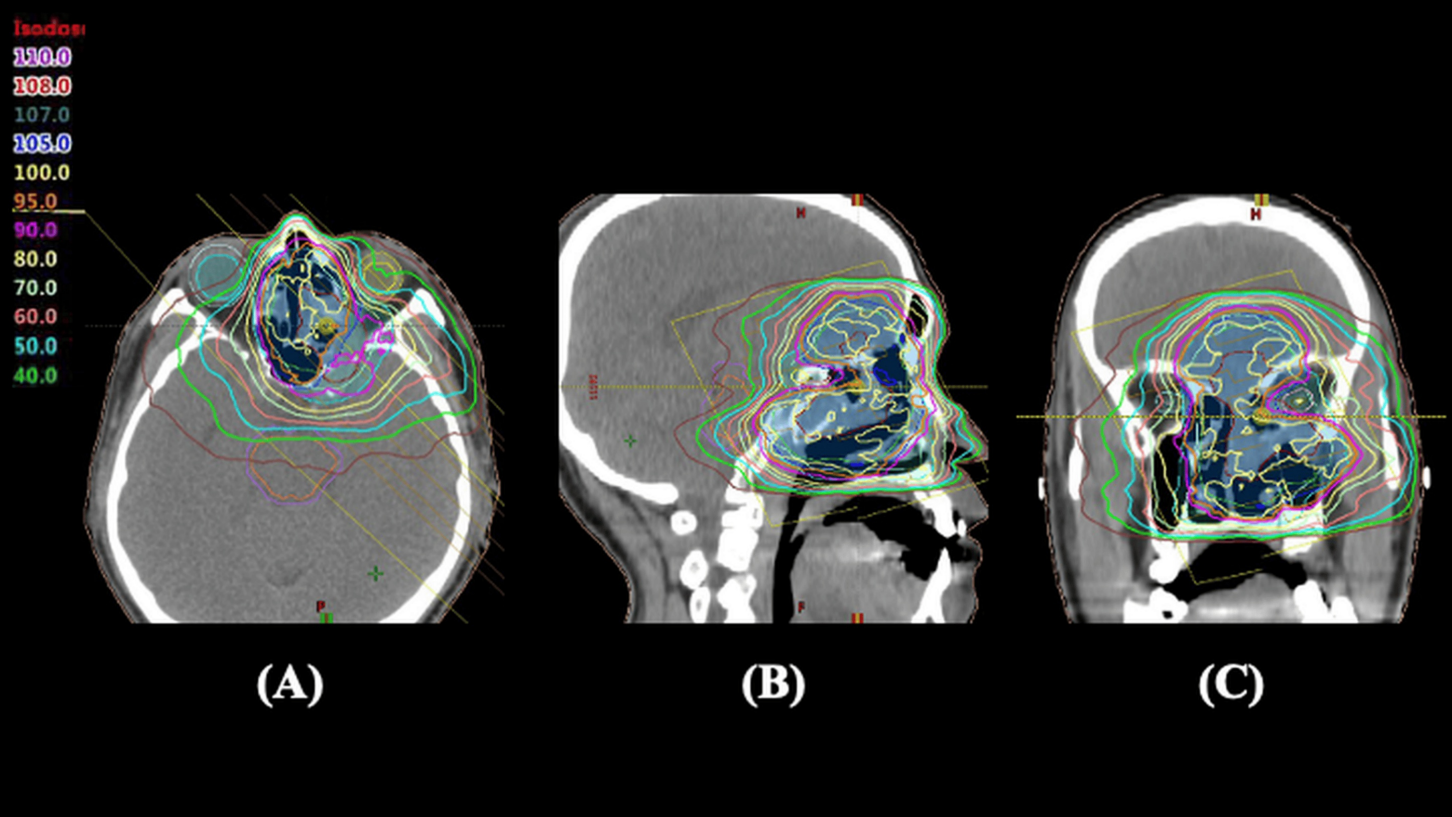
Dose distribution of intensity-modulated radiation therapy (IMRT) using a simultaneous integrated boost (SIB) technique Axial (A), sagittal (B), and coronal (C) CT images show the dose distribution. The gross tumor volume (GTV) is contoured in dark red, the clinical target volume (CTV) in lime green, and the planning target volume (PTV) in cyan. The left eyeball is delineated in yellow. Colored isodose lines represent percentages of the prescribed dose.

Owing to extensive local invasion, surgical resection was deemed infeasible. Therefore, a concurrent chemoradiotherapy (CCRT) strategy was selected. Chemotherapy using the PE regimen (cisplatin 80 mg/m² and etoposide 100 mg/m²) was administered every four weeks for six cycles. After the first cycle of chemotherapy, follow-up CT imaging performed on day 28 revealed partial tumor shrinkage, with a reduction in tumor volume of more than 30% as compared to baseline. This early response supported the initiation of radiation therapy while continuing the chemotherapy regimen. Following this initial response, the patient underwent IMRT comprising 60 Gy in 30 fractions over six weeks. Radiation therapy was delivered using a 10-MV X-ray generator and volumetric modulated arc therapy techniques, with a daily dose of 2.0 Gy administered five times per week. Three radiation volumes were planned: the gross tumor volume (GTV), clinical target volume (CTV), and planning target volume (PTV). GTV was defined based on pre-radiotherapy CT obtained for radiation planning. Since the tumor was too large to spare some surrounding at-risk organs, including the optic nerves, the CTV was defined as an area with a 5-mm margin around the GTV, including the contralateral nasal cavity. Furthermore, PTV was described as an area with a 5-mm margin beyond the CTV. The prescribed dose ensured a minimum dose of at least 95% to the PTV, and inverse optimization was performed. A simultaneous integrated boost (SIB) technique was employed, delivering 66 Gy in 30 fractions (2.2 Gy per fraction) to the GTV and 60 Gy in 30 fractions (2.0 Gy per fraction) to the PTV. Special attention was given to sparing the optic nerve, and the maximum dose to the optic nerve was limited to <54 Gy to minimize the risk of radiation-induced optic neuropathy. Figure 3 shows the dose distribution for this patient.

**Figure 4:**
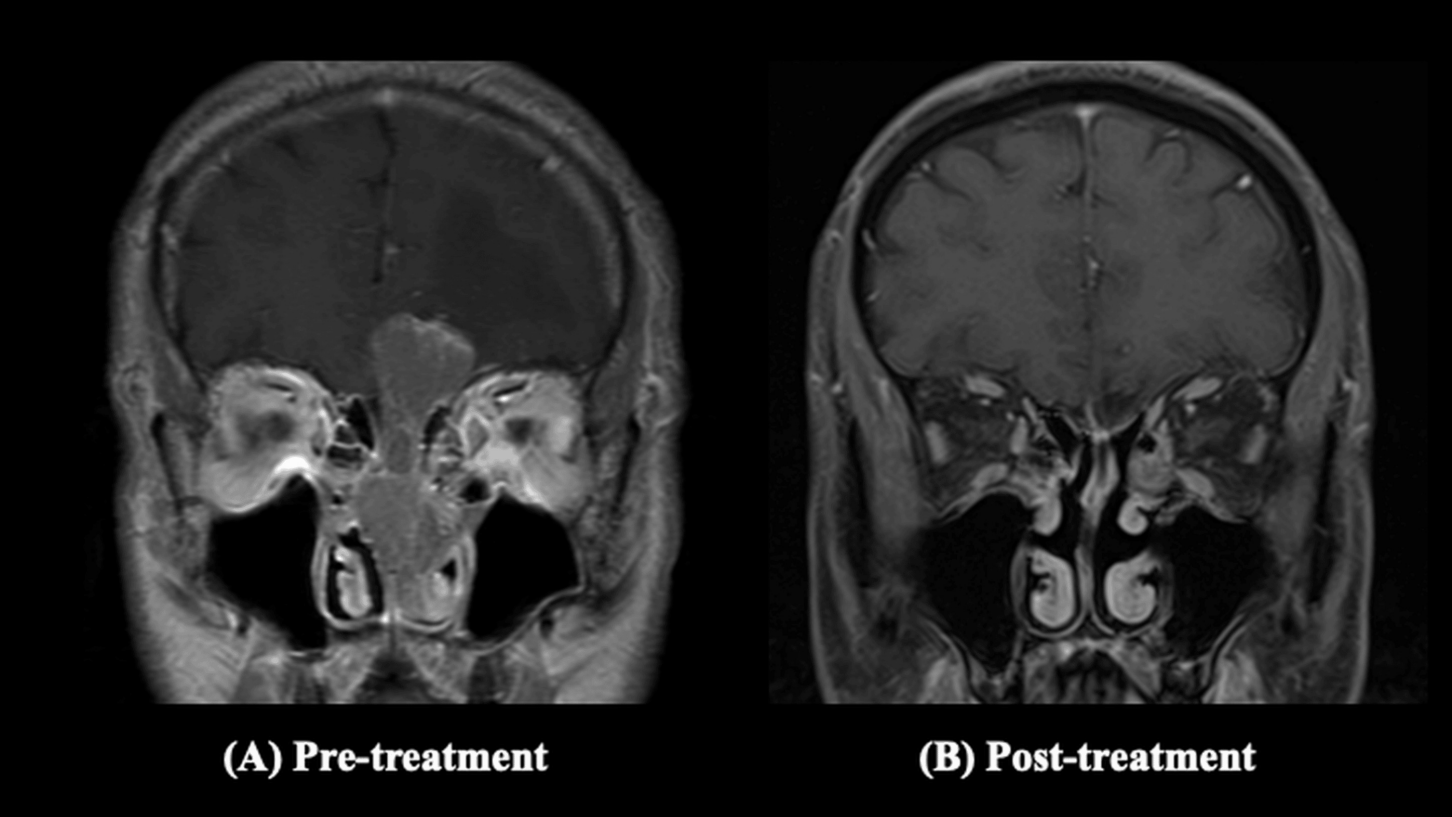
Coronal gadolinium-enhanced T1-weighted magnetic resonance images before and after treatment (A) Pre-treatment MRI shows a strongly enhancing mass occupying the left nasal cavity with superior extension into the anterior cranial base; (B) Post-treatment image demonstrates complete resolution of the enhancing lesion following concurrent chemoradiotherapy.

The treatment was well-tolerated. Adverse events were evaluated using the Common Terminology Criteria for Adverse Events (CTCAE) version 5.0. No grade ≥2 radiotherapy-related adverse events were observed during treatment. The patient experienced mild (grade 1) radiation-induced dermatitis and mucositis, both of which resolved with conservative management. Chemotherapy-related toxicities included transient grade 1 nausea and grade 2 neutropenia, which were managed without treatment delay. No cumulative toxicities or late adverse events, including optic neuropathy or endocrinopathy, were observed during the three-year follow-up. Post-treatment MRI revealed a complete resolution of the tumor (Figure 4). No evidence of recurrence or distant metastasis was detected at the three-year follow-up. The patient also experienced significant visual improvement. Formal ophthalmologic examination demonstrated improvement in best-corrected visual acuity in the left eye to 1.2 × −8.00 D. Although visual field testing was not repeated, the patient subjectively reported complete resolution of the previously noted inferior hemianopia, consistent with clinical observation.

## Discussion

SNUC often presents with nonspecific symptoms, such as nasal obstruction or epistaxis, which can delay diagnosis. Immunohistochemical markers, including CK and CD56, and a high Ki-67 index are critical for accurate diagnosis. Despite advancements in diagnostic and therapeutic modalities, the five-year overall survival rate remains low, ranging from 32% to 38.1%, with a progression-free survival rate of approximately 42% [3,5]. Severe complications associated with high-dose radiotherapy or combination therapy occur in approximately 17% of patients, with acute grade ≥3 toxicities, such as mucositis and dermatitis, being most frequently observed [5].

In our patient, visual-field impairment necessitated advanced imaging and histopathological analysis to facilitate an accurate diagnosis. Surgical resection was infeasible owing to extensive local invasion, underscoring the importance of a multimodal approach. CCRT was used to exploit the synergistic effects of platinum-based chemotherapy and IMRT. Gamez et al. suggested that tertiary therapy, particularly IMRT with prescribed doses >60 Gy, is effective for treating SNUC [8]. The combination of IMRT with the SIB technique enabled precise tumor targeting while minimizing adverse effects on critical structures such as the optic nerve and brainstem.

Remarkably, the patient achieved complete tumor resolution and significant functional recovery, including visual improvement. Formal visual field testing conducted before treatment revealed left inferior quadrantanopia, consistent with optic pathway compression. Follow-up perimetry was performed three months after treatment, and it demonstrated complete resolution of the visual field defect. Vision restoration as a treatment outcome for SNUC has been rarely reported, making this case noteworthy. These outcomes emphasize the importance of precision radiotherapy and individualized treatment planning.

The tolerability of radiotherapy, particularly that of the optic nerve, remains critical. Fraction size and total dose significantly influence the risk of radiation-induced optic neuropathy. Shrieve et al. reported that the tolerance of optic apparatus for single-fraction stereotactic radiosurgery was 8-10 Gy [9]. In contrast, Hasegawa et al. reported that the incidence of optic neuropathy at doses <15 Gy was only 3% among patients undergoing fractionated radiotherapy [10]. However, doses >59 Gy significantly increase the risk of optic neuropathy, particularly with fraction sizes ≥1.9 Gy [11,12]. These findings highlight the need for meticulous dose planning and fractionation. Risk factors for radiation-induced optic neuropathy include a high total radiation dose (>59 Gy), large fraction size (≥1.9 Gy), tumor proximity to critical structures, such as the optic apparatus, and pre-existing vascular comorbidities such as hypertension or diabetes mellitus [9-12].

Although the outcomes in this case are encouraging, SNUC remains a highly challenging disease due to its aggressive clinical behavior and overall poor prognosis. In the context of sinonasal undifferentiated carcinoma, particularly unresectable stage IVB disease, three-year disease-free survival is exceedingly rare. Previous studies have reported a median overall survival of 12-18 months for patients with unresectable SNUC, with 5-year disease-free survival rates of less than 20% in this subgroup [5,13]. Therefore, our case's sustained complete response and functional recovery may represent an unusually favorable outcome within this clinical context. Mauricio et al. reported that distant metastasis remains a significant cause of mortality, highlighting the need for novel treatment strategies [8]. Advances in molecular profiling have identified potential therapeutic targets, including epidermal growth factor receptor and programmed death-ligand 1, offering hope for improved management of refractory or recurrent cases. Additionally, emerging modalities, such as proton therapy, hold promise for enhanced tumor targeting with reduced toxicity. Furthermore, a large-scale analysis of 460 patients using the National Cancer Database by Khan et al. revealed that multimodal therapy, including chemoradiotherapy, was associated with improved overall survival compared to monotherapy, further supporting the role of integrated treatment strategies in SNUC management [13].

## Conclusions

This case illustrates the potential of concurrent chemoradiotherapy with platinum-etoposide and precision IMRT using a simultaneous integrated boost (SIB) to achieve durable tumor control and functional recovery in patients with unresectable sinonasal undifferentiated carcinoma. The favorable outcome - complete response, visual improvement, and three-year disease-free survival - highlights the value of multidisciplinary care and individualized treatment planning. Nevertheless, larger studies and longer follow-ups are necessary to validate these findings and refine optimal management strategies for this aggressive malignancy.
